# Effect of Body Mass Index in Coronary CT Angiography Performed on a 256-Slice Multi-Detector CT Scanner

**DOI:** 10.3390/diagnostics12020319

**Published:** 2022-01-27

**Authors:** Wei-Yip Law, Guan-Lin Huang, Ching-Ching Yang

**Affiliations:** 1 Department of Radiology, Shin Kong Wu Ho-Su Memorial Hospital, Taipei 111, Taiwan; g000@ms.skh.org.tw; 2 Department of Nuclear Medicine, Mennonite Christian Hospital, Hualien 970, Taiwan; s10525006@ems.tcust.edu.tw; 3 Department of Medical Imaging and Radiological Sciences, Kaohsiung Medical University, Kaohsiung 807, Taiwan; 4 Department of Medical Research, Kaohsiung Medical University Chung-Ho Memorial Hospital, Kaohsiung 807, Taiwan

**Keywords:** CCTA, BMI, radiation dose, image quality

## Abstract

We aimed to investigate the effect of a patient’s body mass index (BMI) on radiation dose and image quality in prospectively ECG-triggered coronary CT angiography (CCTA) performed on a 256-slice multi-detector CT scanner. In total, 87 consecutive patients receiving CCTA examinations acquired with tube current modulation (TCM) and iterative reconstruction (IR) were enrolled in this study. The dose report recorded from the CT scanner console was used to derive the effective dose for patients. Subjective image quality scoring and objective noise measurements were conducted to quantify the impact of BMI on the image quality of CCTA. Because of the TCM technique, we expected tube current and radiation dose to increase as BMI increased. However, using TCM did not always guarantee sufficient radiation exposure to achieve consistent image quality for overweight or obese patients since the maximum X-ray tube output in milliamperes and kilovoltage peak was reached. The impact of photon starvation noise on image quality was not significant until BMI ≥ 27 kg/m^2^; this result could be due to IR’s noise reduction capability. Our results also suggest that using TCM with a noise index of 25 HU can reduce radiation dose without compromising image quality compared to images obtained based on the manufacturer’s default settings.

## 1. Introduction

Obesity is a global epidemic, and its prevalence continues to increase in many countries. According to the World Health Organization (WHO) criteria [1], a body mass index (BMI) of 18.5–24.9 kg/m^2^ is considered normal weight, while a BMI of 25–29.9 kg/m^2^ is considered overweight or pre-obese. A BMI ≥ 30 kg/m^2^ is defined as obese, and the obese category can be sub-divided into obese class I (30–34.9 kg/m^2^), obese class II (35–39.9 kg/m^2^), and obese class III (≥40 kg/m^2^). Obesity is a significant risk factor for several subtypes of cardiovascular diseases, such as coronary heart disease, stroke, and heart failure [2,3,4]. Coronary CT angiography (CCTA) is widely recognized as an important imaging tool in the diagnosis of coronary artery disease. However, high photon attenuation and scatter both affect the image quality of CCTA scans of obese patients. The tube current modulation (TCM) system automatically adapts X-ray output according to a patient’s shape, size, and attenuation to achieve a specified image quality with the goal of providing adequate image quality at the lowest possible radiation dose [5,6,7]. In addition to TCM, iterative reconstruction (IR) also has the ability to reduce photon starvation noise resulting from patient attenuation. IR methods are developed to reduce image noise by performing a recursive search for the best estimate to provide an image quality at higher noise levels similar to images acquired by conventional filtered back projection (FBP) [8,9,10,11]. Equivalent quality between images with a dose reduction of 44–54% and FBP images has been reported with the application of IR in CCTA [12,13,14]. Because TCM and IR methods have good dose efficiency to achieve desired image quality, they may prove beneficial to improving the diagnostic accuracy of CCTA by reducing the photon starvation noise that is due to patient attenuation. The aim of this study was to investigate the image quality and radiation dose of prospectively ECG-triggered CCTA acquired with TCM and IR performed on a 256-slice multi-detector CT (MDCT) scanner for patients with BMIs < 25 and ≥25 kg/m^2^.

## 2. Materials and Methods

### 2.1. Study Population

A total of 87 consecutive CCTA examinations for adult patients (26 women and 61 men; age range: 31–82 years; heart rate (HR) range: 45–106 bpm; and BMI range: 18.21–38.19 kg/m^2^) undergoing prospectively ECG-triggered axial scans were investigated in this study. Exclusion criteria for CCTA included known iodine allergy, impaired renal function, and pregnancy. Patients were referred because of chest pain (16%), dyspnea (7%), positive stress test (10%), elevated cardiovascular risk (3%), coronary stent (3%), physical exam (50%), and follow-up (11%). Approval was gained from the Institutional Human Research Ethics Committee to obtain access to patient data.

### 2.2. CCTA Acquisition

Patients with an HR > 70 bpm were given 10–30 mg of oral propranolol (Propranolol Tab, Standard Chem & Pharm Co., Ltd., Tainan, Taiwan) one hour before examination. All patients received 0.6 mg of sublingual nitroglycerin (Nitrostat, Pfizer Pharma, Vega Baja, Puerto Rico) 2 min prior to the scan unless contraindicated. CCTA acquisition was performed using prospectively ECG-triggered axial scanning on a 256-row detector CT scanner (Revolution CT, GE Healthcare, Milwaukee, WI, USA). Non-ionic contrast agent (Optiray 350, loversol injection 74%, Liebel-Flarsheim Canada Inc., Pointe-Claire, QC, Canada) was administered intravenously in a dose of 0.8 mL/kg body weight at a flow rate of 3.5–6.0 mL/s, followed by a 30 mL saline chaser at 3.0–5.0 mL/s. CCTA scans were triggered automatically by a delay of 5.9 s when contrast enhancement within the descending aorta reached a threshold level of 80 Hounsfield units (HU). The scan field of view (SFOV) and *z*-axis coverage were chosen according to the heart size. The ECG pulsing window was set at 70–85% of the R-R interval for patients with an HR ≤ 65 bpm. For patients with 65 bpm < HR ≤ 85 bpm, full tube current was applied at 40–55% and 70–85% of the R-R interval. For patients with an HR > 85 bpm, the ECG pulsing window was set at 20–90% of the R-R interval. The tube voltage was adjusted manually based on patient attenuation, while the tube current was determined automatically by a TCM system (SmartmA, GE Healthcare, Milwaukee, WI, USA) to achieve a noise index (NI) of 22 HU. After scanning, images were reconstructed with 50% FBP and ASiR-V blending, a slice thickness of 0.625 mm, and a 512 × 512 matrix size. Axial images, volume rendering images, and curved planar reformation images were comprehensively evaluated. A cardiac motion correction algorithm (SnapShot Freeze, GE Healthcare, Milwaukee, WI, USA) was used during reconstruction to reduce coronary motion blurring.

### 2.3. Image Quality Evaluation

The image quality of CCTA scans was evaluated by conducting subjective and objective image quality assessments [15,16].

**Figure 1:**
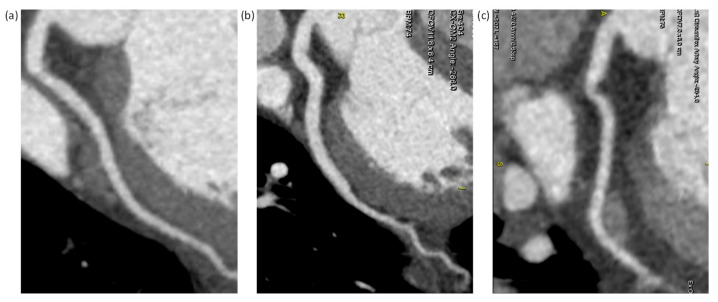
Curved multiplanar reformations of the left circumflex artery (LCX) subjectively rated at 1, 2, and 3 (left to right). (**a**) Heart rate (HR) = 61 bpm, body mass index (BMI) = 21.8 kg/m^2^, and noise index (NI) = 24 HU; (**b**) HR = 74 bpm, BMI = 27.7 kg/m^2^, and NI = 27.7 HU; (**c**) HR = 73 bpm, BMI = 31.1 kg/m^2^, and NI = 30.4 HU.

Subjective image quality was rated using a 4-point grading scale for quantifying the impact of photon starvation noise on every coronary artery segment with a diameter of at least 1.0 mm: 1 = excellent (excellent attenuation of the vessel lumen and clear delineation of the vessel walls with limited perceived image noise); 2 = good (impact of image noise, limitations of low contrast resolution, and minimal vessel margin definition); 3 = adequate (reduced image quality with poor vessel wall definition or excessive image noise, and limitations of low-contrast resolution remained evident); 4 = poor (impaired image quality limited by excessive noise or poor vessel wall definition). Coronary arteries were classified according to the American Heart Association 15-segment model [17]. The right coronary artery (RCA), the left main and left anterior descending artery (LAD), and the left circumflex artery (LCX) were defined to include segments 1–4, 5–10, and 11–15, respectively. Two experienced cardiovascular radiologists rated images by consensus to quantify the degradation of image quality based on photon starvation noise. The subjective image quality scores rated on a per-segment basis were averaged to give an overall score for each vessel. Examples of CCTA images rated at 1 (excellent), 2 (good), and 3 (adequate) are shown in Figure 1. Because no image was rated at 4 (poor) in this study, an example image is not shown. Please refer to [16] for a CCTA image with poor quality.

Objective noise measurements expressed as image noise, contrast-to-noise ratio (CNR), and signal-to-noise ratio (SNR) were obtained by evaluation of the region of interest (ROI) in each image. Image noise was obtained by calculating the standard deviation (SD) of the CT numbers within a contrast-filled left ventricle (circular region: 0.2 cm^2^). CNR was calculated as the difference between the mean signal intensity of the contrast-filled left ventricle and the mean signal intensity of the left ventricular posterolateral wall, divided by the image noise defined previously (circular region: 0.2 cm^2^). SNR was calculated as the mean signal intensity of the contrast-enhanced coronary lumen divided by its SD in the proximal segments of the left and right coronary arteries (circular region: 0.02 cm^2^).

### 2.4. Radiation Dose

The volume CT dose index (CTDI_vol_) and the dose-length product (DLP) displayed on the scanner console were recorded after CCTA scans. The effective dose was calculated as the product of DLP times a conversion coefficient for the chest (k = 0.014 mSv × [mGy × cm]^−1^) [18].

### 2.5. Statistical Analysis

Student’s t-test was used to compare numerical data, including age, BMI, HR, HR variability, tube voltage, tube current, exposure time, SFOV, *z*-axis coverage, CTDI_vol_, DLP, effective dose, contrast dose, image noise, CNR, and SNR. For the subjective image quality scores, which are ordinal data, comparisons were performed using the Mann–Whitney U test. A statistically significant difference was defined as a two-sided *p*-value < 0.05. The inter-observer agreement for subjective image quality scores was analyzed with the kappa test (κ < 0.40: poor agreement; 0.40 ≤ κ < 0.75: good agreement; and κ ≥ 0.75: excellent agreement). Statistical analysis was performed using SPSS version 18 (IBM, Armonk, New York, NY, USA).

## 3. Results

A total of 87 patients were enrolled in this study, including 45 patients with BMIs < 25 kg/m^2^ and 42 patients with BMIs ≥ 25 kg/m^2^. Detailed patient characteristics, scan parameters, and radiation doses are summarized in Table 1. When comparing these two BMI groups, significant differences were found in age, BMI, tube voltage, tube current, CTDI_vol_, DLP, and effective dose.

**Figure 2:**
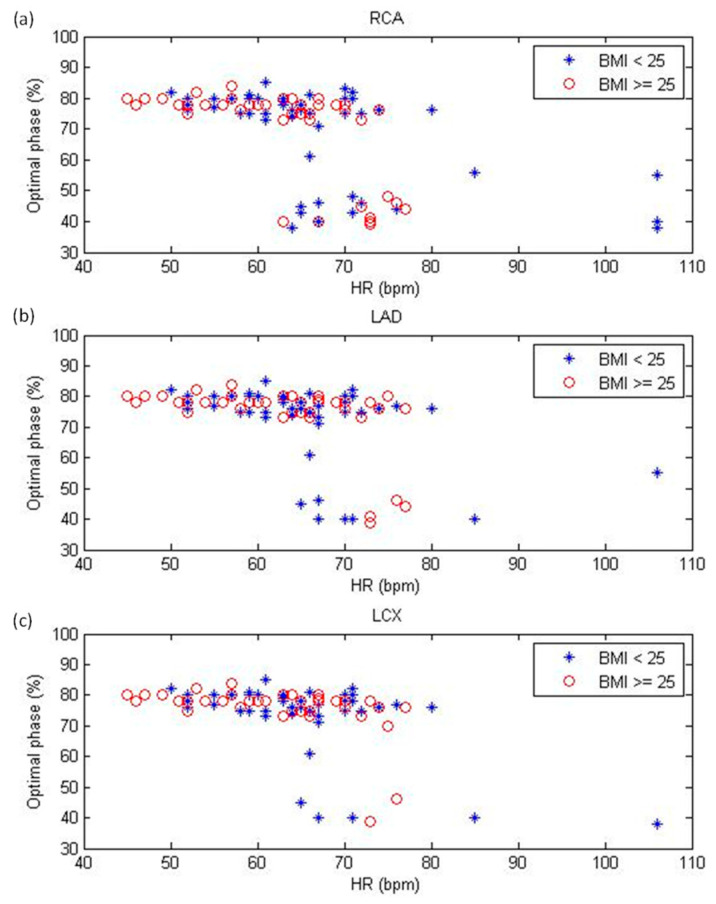
Optimal reconstruction phase for the (**a**) right coronary artery (RCA), (**b**) left anterior descending artery (LAD), and (**c**) left circumflex artery (LCX) as functions of heart rate (HR) on a per-segment basis. BMI = body mass index.

Figure 2 shows the optimal reconstruction phase for the RCA, LAD, and LCX. There were 1098 coronary artery segments in 87 patients, which included 338 RCA segments, 491 LAD segments, and 269 LCX segments. Systolic and diastolic reconstruction were used in 20.41% and 79.59% of the RCA segments, respectively (BMI < 25 kg/m^2^: 38 segments vs. 140 segments; BMI ≥ 25 kg/m^2^: 31 segments vs. 129 segments). The corresponding results were 7.74% and 92.26% for LAD segments (BMI < 25 kg/m^2^: 24 segments vs. 233 segments; BMI ≥ 25 kg/m^2^: 14 segments vs. 220 segments) and 7.06% and 92.94% for LCX segments (BMI < 25 kg/m^2^: 13 segments vs. 124 segments; BMI ≥ 25 kg/m^2^: 6 segments vs. 126 segments).

**Figure 3:**
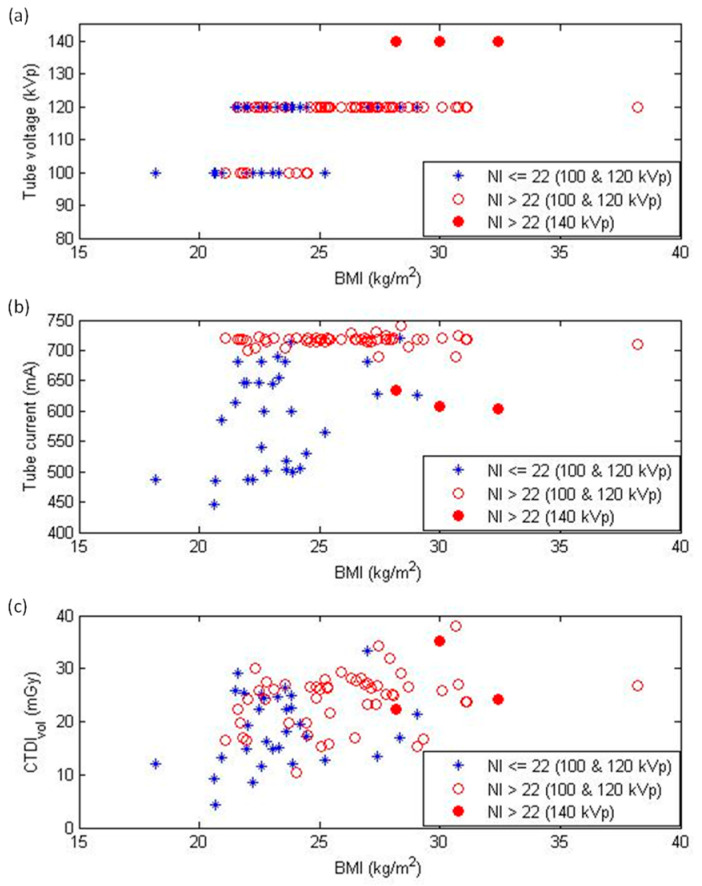
Distribution of (**a**) tube voltage, (**b**) tube current, and (**c**) volume CT dose index (CTDI_vol_) as functions of body mass index (BMI). NI = noise index.

Table 2 summarizes the contrast agent doses, scan parameters, and radiation doses in different BMI groups. These baseline characteristics significantly increased for BMIs of 25–26, 27–28, and >28 kg/m^2^ compared to a BMI < 23 kg/m^2^. Figure 3 demonstrates the distribution of tube voltage, tube current, and CTDI_vol_ as the functions of BMI.

**Figure 4:**
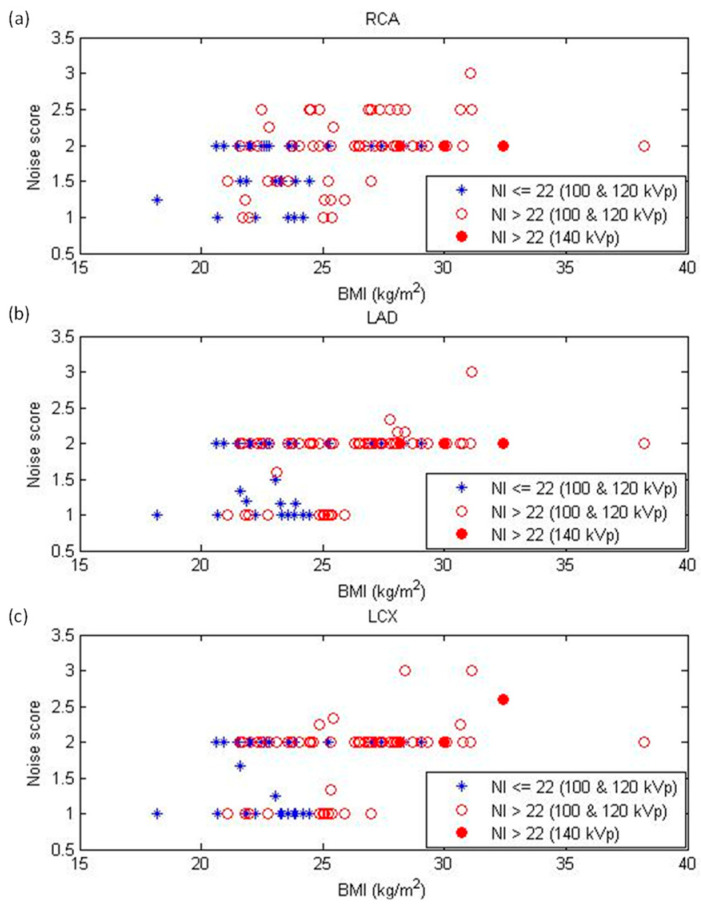
Subjective noise score on a per-vessel basis for the (**a**) right coronary artery (RCA), (**b**) left anterior descending artery (LAD), and (**c**) left circumflex artery (LCX) as functions of body mass index (BMI). NI = noise index.

**Figure 5:**
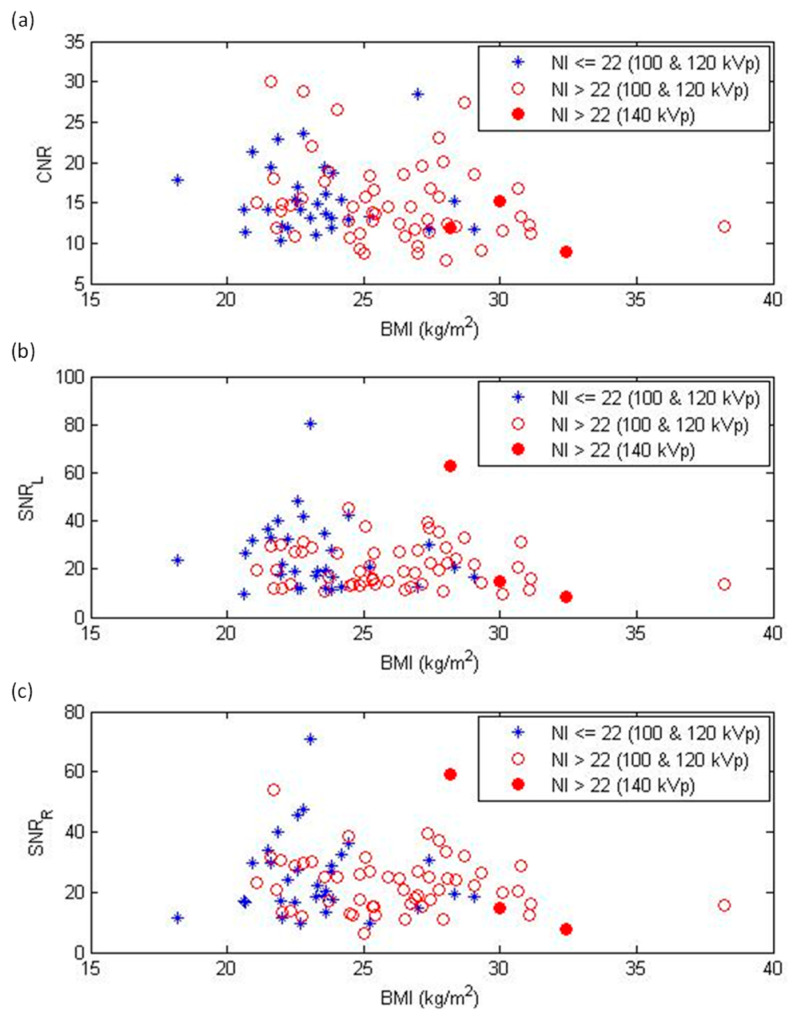
Objective image quality expressed in terms of (**a**) contrast-to-noise ratio (CNR), (**b**) signal-to-noise ratio of left coronary artery (SNR_L_), and (**c**) signal-to-noise ratio of right coronary artery (SNR_R_) as functions of body mass index (BMI). NI = noise index.

**Figure 6:**
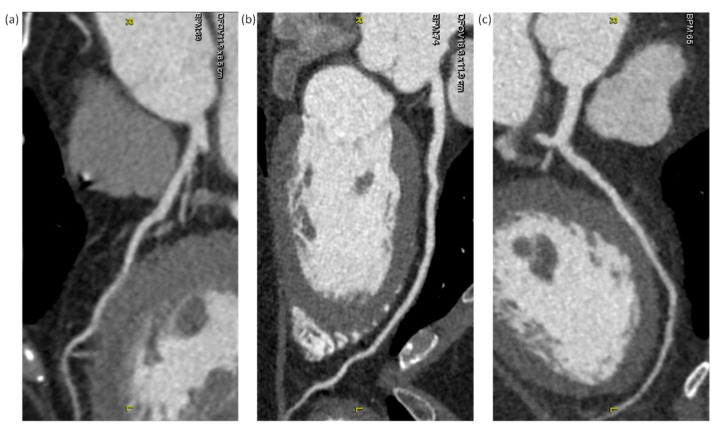
Curved multiplanar reformations of the left anterior descending artery (LAD) subjectively rated at 2. (**a**) Heart rate (HR) = 69 bpm, body mass index (BMI) = 21.6 kg/m^2^, and noise index (NI) = 22.7 HU; (**b**) HR = 74 bpm, BMI = 27.4 kg/m^2^, and NI = 27.7 HU; (**c**) HR = 65 bpm, BMI = 38.2 kg/m^2^, and NI = 38.8 HU.

Table 3 summarizes the NI, subjective image quality scores, and objective noise measurements in different BMI groups. The inter-observer agreement for subjective scores was excellent (kappa = 0.92; 95% confidence interval: 0.86–0.98). Through evaluating these image quality indicators, we found that image noise increased as BMI increased. In comparison with a BMI < 23 kg/m^2^, NI was significantly higher in BMIs of 25–26, 27–28, and > 28 kg/m^2^, while subjective scores significantly degraded in BMIs of 27–28 and > 28 kg/m^2^. For objective noise measurements, statistically significant differences were found in CNR (BMI > 28 kg/m^2^) and SNR of the left and right coronary arteries (SNR_L_: BMI > 28 kg/m^2^; SNR_R_: BMIs of 25–26, > 28 kg/m^2^) when compared to a BMI < 23 kg/m^2^, but not for image noise. Figure 4 demonstrates the subjective scores for the RCA, LAD, and LCX as the functions of BMI. Figure 5 demonstrates the CNR, SNR_L_, and SNR_R_ as functions of BMI. Case examples with a subjective score of two are shown in Figure 6.

## 4. Discussion

With increasing patient thickness, photon attenuation increases exponentially [19]. The resultant artifacts are worse with the use of a low-power generator system, low peak tube voltage, and fast rotation time. To provide consistent image quality for all patients, the TCM technique was used to compensate for the photon starvation noise that is due to patient attenuation. In the TCM system we operated, the NI, or the predefined image quality index, was set at 22 HU, according to the manufacturer’s recommendation. However, an NI > 22 HU indicated that the photon starvation noise was not fully compensated for at this setting. Therefore, we selected a proper tube voltage setting before data acquisition to achieve the desired noise level according to the NI displayed on the console. In this study, CCTA scans were acquired at 100 or 120 kVp for BMI < 25 kg/m^2^, while a tube voltage of 120 or 140 kVp was used for BMI ≥ 25 kg/m^2^ (Figure 3a). Based on our experience, the difference between 120 and 140 kVp CCTA scans when imaging obese patients is small in terms of radiation dose and image noise (Figure 3, Figure 4 and Figure 5). This phenomenon occurs because the maximum tube current is lower at 140 kVp than at 120 kVp. For the CT scanner investigated in this study, the maximum tube current was 740 mA at 100 and 120 kVp, while the maximum tube current was 635 mA at 140 kVp. Additionally, prior studies have shown that the iodine contrast attenuation is lower in CCTA images acquired with a higher kilovoltage peak [20,21,22], so the 140 kVp scan is usually not used in our clinical routine practice.

TCM adjusts the tube current based on the patient attenuation in the scout image to achieve the desired image quality at the lowest dose setting, so there should be a positive correlation between tube current and patient attenuation. However, the positive correlation between tube current and patient attenuation reached a plateau beyond the maximum X-ray output in milliamperes and kilovoltage peak, and the efficacy of TCM on compensating photon starvation noise was limited in the plateau region. As shown in Table 1, we found that kilovoltage peak, milliamperes, CTDI_vol_, DLP, and effective dose are significantly higher for BMI ≥ 25 kg/m^2^. A more detailed comparison shown in Table 2 reveals that these baseline characteristics increased as BMI increased, except the tube current for a BMI of 27–28 k and >28 kg/m^2^. As seen in Figure 3b, there was a positive correlation between tube current and BMI for CCTA scans with an NI ≤ 22 HU (blue stars). However, an increase in BMI did not lead to obvious variation in the tube current of CCTA scans with an NI > 22 HU (red circles). There were 40.0% and 88.1% CCTA scans with an NI > 22 HU for BMI < 25 and ≥25 kg/m^2^, respectively. Because of the small size and rapid motion of the coronary arteries, CCTA requires high spatial resolution (small voxel size) and high temporal resolution (short rotation time) to achieve reliable diagnostic image quality. Consequently, the TCM-adjusted tube current is usually higher for CCTA than in other CT studies, which makes it easier to reach the maximum X-ray output when imaging obese patients. In addition to the TCM modulation range, the photon starvation noise cannot be fully eliminated, and the noise level increases with increasing BMI. Therefore, the CCTA scans acquired using the maximum X-ray output have an NI value larger than 22 HU.

Since the tube voltage, tube current, and radiation doses increased as BMI increased (Table 2 and Figure 3), it is natural to expect that these CCTA scans would provide consistent image quality for all patients. However, we found that the subjective image quality scores and objective noise measurements only slightly increased with increasing BMI (Table 3, Figure 4). This phenomenon could be due to the CCTA scans with an NI > 22 HU. The resulting impact was more serious for patients with BMI ≥ 25 kg/m^2^. Although our results demonstrated a positive correlation between image quality degradation and BMI, the overall subjective score of each vessel was less than three, indicating the image quality was sufficient for clinical diagnosis. As seen in Table 3, the difference between BMI < 23 and BMI 25–26 kg/m^2^ was statistically significant in NI, but not in subjective scoring or objective noise measurements. These results indicated that NI is more sensitive to the variation in BMI than other image quality indicators. By definition, NI is the noise in the central region of a CT image reconstructed with FBP for a 20 cm uniform water phantom [23]. The subjective scoring and objective noise measurements were performed on IR-reconstructed images. IR yields lower image noise and higher spatial resolution than FBP, which could explain why the subjective scoring and objective noise measurements were less sensitive to the increased image noise that is due to patient attenuation compared to NI. Moreover, the subjective scoring and objective noise measurements were defined for contrast-filled vessels and chambers, while NI was defined on a phantom-simulating soft tissue. It was reported that image noise has a larger detrimental effect on the detection of a low-contrast target than it does on detection of a high-contrast target [24]. Therefore, the definition of image quality metrics might be another potential explanation of the differing sensitivity to image noise.

In this study, the CTDI_vol_ for BMI < 25 and ≥25 kg/m^2^ were 20.03 and 24.83 mGy, respectively, which are consistent with previous reports [25,26]. Over the past decades, various dose reduction strategies have been proposed for CCTA scans [27,28]. Some are general techniques (such as prospective ECG triggering, low kilovoltage peak, and TCM) and others are technology-specific techniques (such as hybrid/statistical IR, model-based IR, and deep learning IR). Although minimizing the radiation dose is always an important goal in the CT imaging community, it is also important to ensure sufficient image quality for clinical diagnosis at the same time, i.e., the as low as reasonably achievable (ALARA) principle. Since there was no significant difference in the subjective image quality scores and objective noise measurements between BMI < 23 and 25–26 kg/m^2^, we expected that using an NI of 25 HU could reduce the radiation dose without compromising the image quality for patients receiving CCTA performed on the 256-slice MDCT investigated in this study. Several limitations to this study need to be acknowledged. First, we relied on data that already existed in our department. To avoid selection bias, data were enrolled consecutively during a specific time period, and image analysis was performed without information about BMI. Second, since we focused on the effect of BMI on the radiation dose and image noise of CCTA, the diagnostic performance of CCTA was not compared to invasive catheter angiography. Further studies are necessary to evaluate the impact of BMI on the diagnostic assessment of the coronary arteries with CT. Third, all data were acquired using TCM and IR developed by a single manufacturer. It was difficult to compare our results with other types of TCM and IR techniques from different manufacturers.

## 5. Conclusions

In this study, we investigated the effect of a patient’s BMI on the radiation dose and image quality in prospectively ECG-triggered CCTA performed on a 256-slice MDCT. Because of the TCM technique, we expected that the tube current and radiation dose would increase as BMI increased. However, using TCM could not always guarantee sufficient radiation exposure to achieve consistent image quality for overweight or obese patients since the maximum X-ray tube output in milliamperes and kilovoltage peak was reached. The impact of photon starvation noise on image quality was not significant until BMI ≥ 27 kg/m^2^, which could have been due to IR’s noise reduction capability. Our results also suggest that using TCM with an NI of 25 HU could reduce the radiation dose without compromising image quality compared to images obtained based on the manufacturer’s default settings.

## Figures and Tables

**Table 1:** Comparison of patient characteristics, scan parameters, and radiation doses between two BMI groups.

		BMI < 25	BMI ≥ 25	*p*-Value
Number of patients		45	42	-
Age		60.80 ± 8.75	56.64 ± 10.67	0.049 *
BMI (kg/m^2^)		22.74 ± 1.33	27.93 ± 2.47	<0.001 *
SFOV	small	0	0	0.583
	medium	39	38	
	large	6	4	
*Z*-axis coverage	12 cm	0	2	0.293
	14 cm	18	18	
	16 cm	27	22	
HR (bpm)		65.16 ± 9.55	62.71 ± 8.61	0.215
HR variability (bpm)		5.71 ± 4.72	5.57 ± 3.28	0.874
Tube voltage (kVp)		112.44 ± 9.81	120.95 ± 6.17	<0.001 *
Tube current (mA)		637.38 ± 92.17	701.67 ± 39.94	<0.001 *
Exposure time (s)		0.74 ± 0.09	0.71 ± 0.11	0.259
CTDI_vol_ (mGy)		20.03 ± 6.22	24.83 ± 5.78	<0.001 *
DLP (mGy × cm)		303.49 ± 96.21	372.89 ± 90.84	0.001 *
Effective dose (mSv)		4.25 ± 1.35	5.22 ± 1.27	0.001*

* Statistically significant difference was found between the 2 BMI groups. Note: BMI = body mass index, SFOV = scan field of view, HR = heart rate, CTDI_vol_ = volume CT dose index, DLP: dose-length product.

**Table 2:** Comparison of contrast agent doses, scan parameters, and radiation doses in different BMI groups.

	BMI (kg/m^2^)	Number of Patients	Contrast Agent (mL)	Tube Voltage (kVp)	Tube Current (mA)	CTDI_vol_ (mGy)	Effective Dose (mSv)
BMI < 25	<23	25	55.28	111.20	627.56	19.42	4.13
	23–24	20	60.45 (*p* = 0.180)	114.00 (*p* = 0.347)	649.65 (*p* = 0.431)	20.80 (*p* = 0.467)	4.40 (*p* = 0.506)
BMI ≥ 25	25–26	17	68.47 * (*p* < 0.001)	118.82 * (*p* = 0.002)	707.82 * (*p* = 0.001)	24.32 * (*p* = 0.022)	5.09 * (*p* = 0.040)
	27–28	14	73.14 * (*p* < 0.001)	121.43 * (*p* < 0.001)	705.71 * (*p* = 0.001)	25.10 * (*p* = 0.012)	5.20 * (*p* = 0.031)
	>28	11	82.36 * (*p* < 0.001)	123.64 * (*p* = 0.001)	687.00 * (*p* = 0.02)	25.27 * (*p* = 0.026)	5.44 * (*p* = 0.021)

* Statistically significant difference was found when compared to BMI < 23 kg/m^2^. Note: BMI = body mass index, CTDI_vol_ = volume CT dose index.

**Table 3:** Comparison of NI, subjective image quality score, and objective noise measurements in different BMI groups.

	BMI (kg/m^2^)	Number of Patients	NI (HU)	Noise Score			Image Noise (HU)	CNR	SNR_L_	SNR_R_
				RCA	LAD	LCX				
BMI < 25	<23	25	22.82	1.73	1.66	1.67	26.40	16.57	25.15	25.44
	23–24	20	23.56 (*p* = 0.237)	1.75 (*p* = 0.689)	1.57 (*p* = 0.808)	1.58 (*p* = 0.735)	27.57 (*p* = 0.636)	15.19 (*p* = 0.346)	24.00 (*p* = 0.779)	25.49 (*p* = 0.990)
BMI ≥ 25	25–26	17	25.68 * (*p* < 0.001)	1.76 (*p* = 0.460)	1.65 (*p* = 0.739)	1.63 (*p* = 0.755)	29.45 (*p* = 0.272)	14.24 (*p* = 0.148)	19.92 (*p* = 0.080)	18.16 * (*p* = 0.018)
	27–28	14	28.11 * (*p* < 0.001)	2.18 * (*p* = 0.002)	2.05 * (*p* = 0.006)	2.07 * (*p* = 0.005)	27.88 (*p* = 0.628)	15.59 (*p* = 0.582)	28.77 (*p* = 0.348)	27.82 (*p* = 0.564)
	>28	11	30.64 * (*p* = 0.002)	2.18 * (*p* = 0.011)	2.09 * (*p* = 0.008)	2.171 * (*p* = 0.014)	30.93 (*p* = 0.133)	12.79 * (*p* = 0.033)	16.29 * (*p* = 0.004)	18.46 * (*p* = 0.028)

*: Statistically significant difference was found compared to BMI < 23 kg/m^2^. Note: BMI = body mass index, NI = noise index, RCA = right coronary artery, LAD = left anterior descending artery, LCX = left circumflex artery, CNR = contrast-to-noise ratio, SNR_L_ = signal-to-noise ratio of left coronary artery, SNR_R_ = signal-to-noise ratio of right coronary artery.

## Data Availability

The authors confirm that the data supporting the findings of this study are available within the article.
